# The complete chloroplast genome and phylogenetic analysis of *Elaeagnus oldhamii* (Elaeagnaceae) from Fujian, southeastern China

**DOI:** 10.1080/23802359.2024.2305399

**Published:** 2024-01-18

**Authors:** Yuan Chen, Ying Zheng, Gongning Shi, Pengfei Wang, Yanxiang Lin, Mingqing Huang, Yanfang Zheng

**Affiliations:** a College of Pharmacy, Fujian University of Traditional Chinese Medicine, Fuzhou, China; b Technology Center, China Tobacco Henan Industrial Co., Ltd, Zhengzhou, China

**Keywords:** *Elaeagnus oldhamii*, plastome, medicinal plant, phylogeny

## Abstract

*Elaeagnus oldhamii* Maximowicz 1870 is an important medicinal plant mainly distributing in the southeastern coastal region of China. However, the complete chloroplast genome of *E. oldhamii* has never been studied at present. We obtained the complete chloroplast genome of *E. oldhamii*, which was 152,283 bp in length, with a typical quadripartite structure that includes a large single-copy region of 82,229 bp, a small single-copy region of 18,256 bp, and 2 inverted repeat (IR) regions of 25,899 bp. The genome contained 128 unique genes with a GC content of 37%, including 83 protein-coding genes, 37 tRNAs, and 8 rRNAs. Phylogenetic analysis suggested that *E. oldhamii* was closely related to *E. glabra* and *E. macrophylla*.

## Introduction

*Elaeagnus oldhamii* Maximowicz 1870, a member of Elaeagnaceae family, is an evergreen upright shrub distributing along the southeastern coast of China (Sun and Lin 2010). As a traditional medicine, recent studies have demonstrated that the compounds contained in *E. oldhamii* possess anti-inflammatory, anti-tumor and analgesic properties, suggesting potential therapeutic applications for human diseases such as rheumatoid arthritis, cataracts, and neoplastic conditions (Liao et al. 2013).

Additionally, *E. oldhamii* is also a vital component of the forest biomes along the coastal area, playing an important role in water and soil conservation. However, the habitat of *E. oldhamii* is under threat due to industrial development and agricultural cultivation, resulting in a significant decline in its population.

Chloroplast genomes, housing a portion of the genetic information in plant cells, are widely used in plant species identification, genetic improvement, and conservation efforts. Research on chloroplast genomes plays a vital role in enhancing our understanding of plant evolution, classification, and genetic diversity, with significant implications for species conservation.

Our objectives were to explore the characteristics of the *E. oldhamii* chloroplast genome, clarify its phylogenetic relationship, and establish the groundwork for future applications of chloroplast genome research in the conservation and genetic breeding of *E. oldhamii*.

## Materials and methods

**Figure 1.**
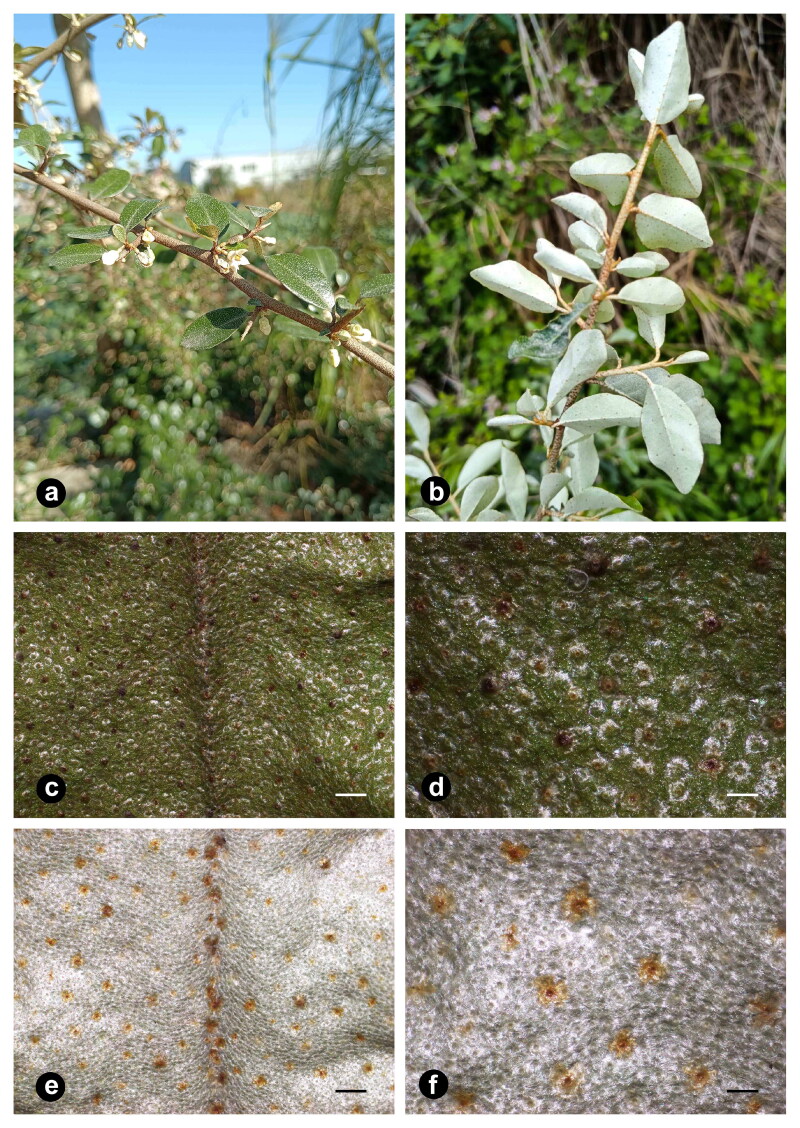
The morphological features of *Elaeagnus oldhamii*. As the most characteristic feature of identifying *Elaeagnus* species, the leaves, young branches and buds of *E. oldhamii* were covered with silver or rusty scales on both sides under the microscope’s field of view. (a) Individual of *E. oldhamii* in natural habitat. The branches densely covered with brown or rusty scales. And the flowers appeared pale white, covered with scales, and several flowers clustered into racemes with short branches in the leaf axils. (b) The leaves were nearly papery, obovate, with cuneate base and obtuse apex. (c) Stereomicrographs of adaxial surface of *E. oldhamii* leaves. The leaf surface was densely covered with silvery scales. (d) Magnified view of the adaxial surface. The scales partially fall off when mature and were slightly shiny and brown. (e) Stereomicrographs of abaxial surface of *E. oldhamii* leaves. The abaxial surface of the leaves was densely covered with silver scales. (f) Magnified view of the abaxial surface. There were a few dark brown scales scattered on the underside of the leaves. Photographs a-b were taken by Yanxiang Lin in Lianjiang County, Fujian Province, and stereomicrographs c-f were taken by yuan Chen at the laboratory of Fujian University of Traditional Chinese Medicine. Bars = 1 mm (c and e) and 400 μm (d and f)

Fresh leaves of *E. oldhamii* were collected from Lianjiang County in Fuzhou, Fujian, China (N 26°20′26.53″, E 119°40′50.38″) (Figure 1). The voucher specimen (No. CP 20230402001) was deposited in the Herbaria of Fujian University of Traditional Chinese Medicine (Contact: Yanxiang Lin, linyanxiang@fjtcm.edu.cn). The genomic DNA was extracted using DNA Quick Plant System (TIANGEN BIOTECH Co., Ltd, Beijing, China). The isolated genomic DNA was utilized to constructed a DNA library and the paired-end reads of 150 bp were generated by the Illumina NovoSeq 6000 sequencing platform (Novogene Bioinformatics Technology Co., Ltd, Tianjin, China). A total of approximately 5.4 GB short sequences were obtained. Fastp v0.23.1 was used to filter the raw sequencing low-quality reads (Chen et al. 2018). Next, the genome sequence was assembled employing GetOrganelle v1.7.6.1 software (Jin et al. 2020). The assembled *E. oldhamii* chloroplast genome was annotated with CPGAVA2 (Shi et al. 2019) and GeSeq v2.03 (Tillich et al. 2017) using the chloroplast genomes of *E. umbellata* (LC522506) as reference sequences. Annotation results were manually checked and confirmed when necessary. The final sequence and annotation file was summitted to NCBI (Accession number: OQ948125). The circular chloroplast genome map of *E. oldhamii* was drawn using Chloroplot (Zheng et al. 2020) and the cis and trans splicing genes were identified using CPGView software (Liu et al. 2023). CPJSdraw software (Li et al. 2023) was used to display the junction sites information in four regions of the chloroplast genome boundaries of *E. umbellata* (LC522506), *E. multiflora* (LC522136), *E. glabra* (MN306572), *E. macrophylla* (NC_028066) and *E. oldhamii*. Additionally, a comparison of the five genomes mentioned above using the Shuffle-LAGAN mode in mVISTA (Frazer et al. 2004).

In order to investigate the phylogenetic relationship of *E. oldhamii* in the family Elaeagnaceae, complete chloroplast genome sequences of other 13 species were collected from NCBI (9 species of *Elaeagnus* and 4 species of *Hippophae*). *Barbeya oleoides* (NC_040984) was used as an outgroup. Fifteen complete chloroplast genome sequences, including that of *E. oldhamii*, were aligned using MAFFT v7.515 (Rozewicki et al. 2019) to construct phylogenetic trees and determine the taxonomic status of *E. oldhamii*. The best-fit model, based on the Bayesian information criterion (BIC), was GTR + F + I + G4. The maximum-likelihood (ML) tree was generated using IQ-TREE v2.2.0.3 (Nguyen et al. 2015) with ultrafast bootstrap (UFBoot) of 10,000 replicates.

## Results

**Figure 2.**
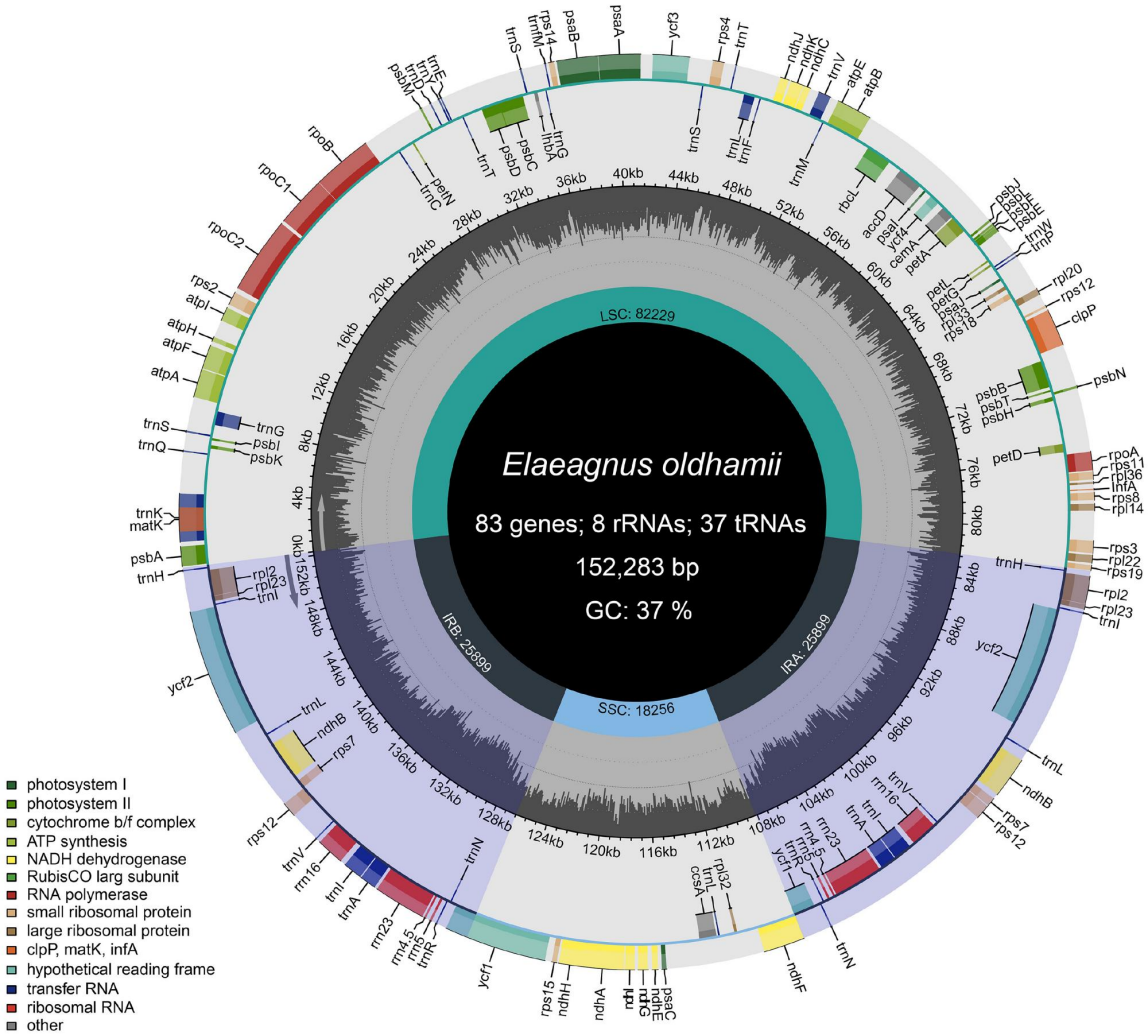
Chloroplast genome map of *E. oldhamii* generated by Chloroplot. The genes inside the circle are transcribed clockwise, and the genes outside the ring are transcribed counterclockwise. The gene functional groups are distinguished using different colors. A pair of IR regions symmetrically separate the LSC (large single Copy) and SSC (small single Copy) regions. Within the circle, the dark grey portion represents the GC content, and the light gray area represents the at content. The functional classification of genes is displayed in the bottom left corner.

The clean reads were mapped to chloroplast genome sequence with an average coverage depth of 1700.76× (Figure S1), indicating that the chloroplast genome of *E. oldhamii* was assembled correctly (https://doi.org/10.17504/protocols.io.4r3l27jkxg1y/v1) (Ni et al. 2023). The complete chloroplast genome of *E. oldhamii* was obtained, with a length of 152,283 bp and a guanine-cytosine ratio is 37% (Figure 2). The genome has a typical quadripartite structure, containing a large single-copy (LSC) region of 82,229 bp, a small single-copy (SSC) region of 18,256 bp, and a pair of inverted-repeat regions (IRs) of 25,899 bp. A total of 128 unique genes were annotated, comprising 83 protein-coding genes (PCGs), 8 ribosomal RNA genes (rRNAs), and 37 transfer RNA genes (tRNAs). Among them, 15 genes (*atpF*, *ndhA*, *rpoC1*, rpl2*2, ndhB*2, trnA-UGC*2, *trnG-GCC*, trnI-GAU*2, *trnK-UUU*, *trnL-UAA*, *trnV-UAC*) are single-intron genes, and 2 genes (*ycf3*, *clpP*) contain two introns. 19 genes (*ndhB*, *rpl2*, *rpl23*, *rps7*, *rps12*, *ycf1*, *ycf2*, *trnA-UGC*, *trnH-GUG*, *trnI-CAU*, *trnI-GAU*, *trnL-CAA*, *trnN-GUU*, *trnR-ACG*, *trnV-GAC*, *rrn16*, *rrn23*, *rrn4.5*, *rrn5*) are duplicated in IR regions.

In terms of chloroplast genome boundaries, the boundary between the IRb and SSC contained the *ycf1* gene, with *ycf1* pseudogenes present in chloroplasts of all five species. The gene length and location of *rps19*, *ndhF*, *trnN* and *trnH* were consistent in *E. oldhamii*, *E. macrophylla* and *E. glabra*, with slight differences compared to *E. umbellata* and *E. multiflora*. The variation in the length of the IR and SSC regions among different species can be attributed to the expansion and contraction of the *ycf1* and *psbA* genes (Figure S2). In our analysis, the variation degree of non-coding region is significantly higher than that of coding region (Figure S3). The LSC region displayed the highest degree of variation, whereas the IR region exhibited the lowest degree of variation with high conservation. There were also variations in certain genes, such as in the *trnK-UUU-psbK*, *rpoB-psbM* and *rps4-ndhJ* regions. Additionally, we detected 9 cis-splicing genes (*atpF*, *rpoC1*, *ycf3*, *clpP*, rpl2*2, ndhB*2, *ndhA*) and only one trans-splicing genes (*rps12*) (Figure S4).

**Figure 3.**
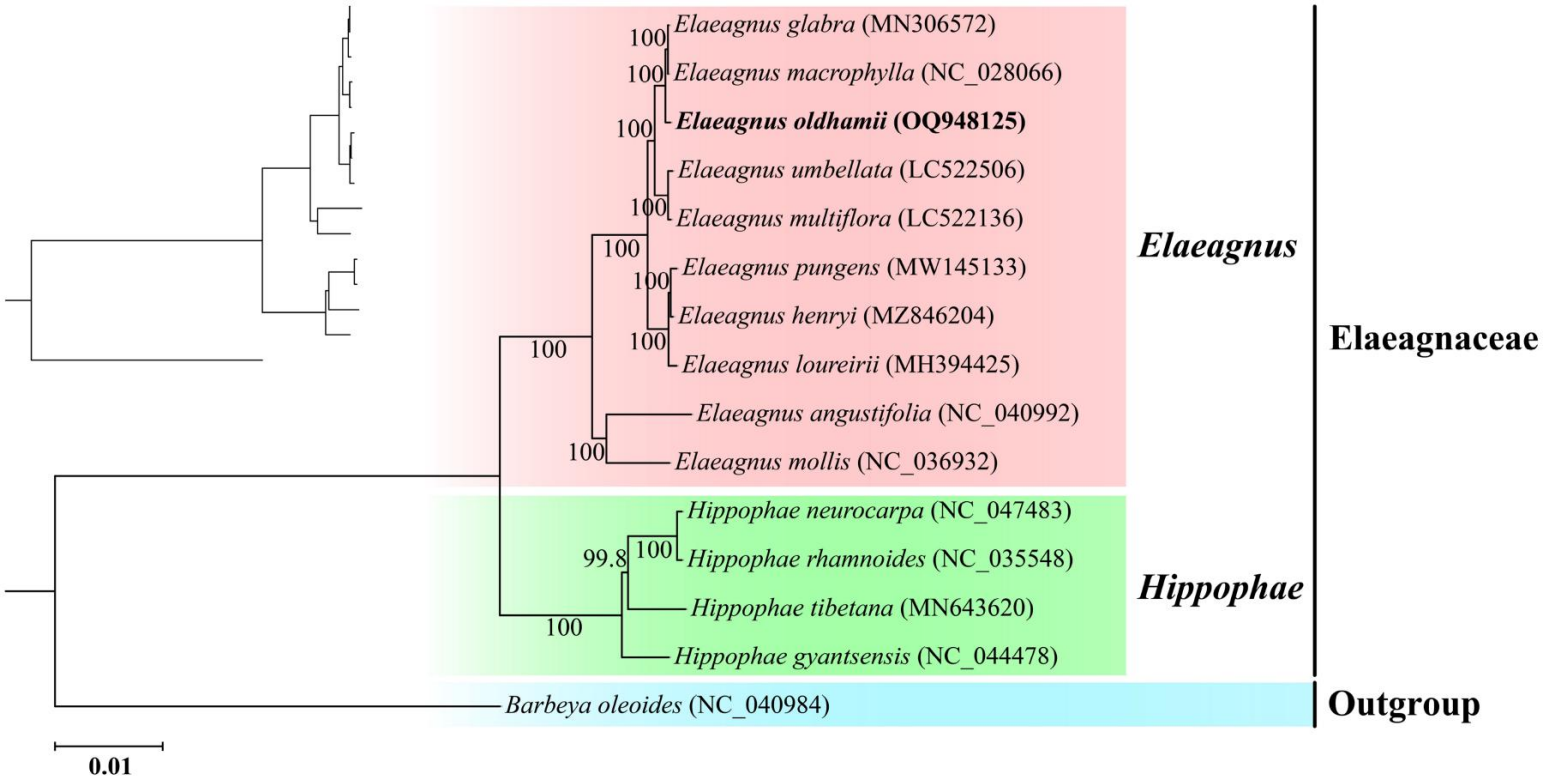
Maximum-likelihood phylogenetic tree of 10 species of *Elaeagnus* genus and 4 species of *Hippophae* genus based on complete chloroplast genome sequences. The maximum-likelihood (ML) tree was generated using IQ-TREE v2.2.0.3 (Nguyen et al. 2015) with ultrafast bootstrap (UFBoot) of 10,000 replicates. The best-fit model, based on the Bayesian information criterion (BIC), was GTR + F + I + G4. The numbers above branches indicate ultrafast bootstrap support (%) value of maximum-likelihood. The following sequences were used: *E. glabra* (MN306572) (Zhao et al. 2020), *E. macrophylla* (NC_028066) (Choi et al. 2015), *E. umbellata* (LC522506) (Kim et al. 2020), *E. multiflora* (LC522136) (Kim et al. 2020), *E. pungens* (MW145133) (Lu et al. 2022), *E. henryi* (MZ846204) (Chang et al. 2022), *E. loureirii* (MH394425) (Zeng et al. 2018), *E. angustifolia* (NC_040992), *E. mollis* (NC_036932) (Cheng et al. 2020), *Hippophae neurocarpa* (NC_047483) (Zhou et al. 2019), *H. rhamnoides* (NC_035548) (Chen and Zhang 2017), *H. tibetana* (MN643620) (Zhou et al. 2020), *H. gyantsensis* (NC_044478) (Wang et al. 2021), *Barbeya oleoides* (NC_040984).

The phylogenetic analysis reveals that the chloroplast genomes of *E. oldhamii* was clustered with *E. glabra* and *E. macrophylla* chloroplast genomes, and formed a monophyletic group with other *Elaeagnus* species by high supportive values (Figure 3). The phylogenetic outcomes of this study are consistent with those previously reported by Chang et al. (Chang et al. 2022), strengthening the current understanding of the evolutionary relationships of this genus.

## Discussion and conclusions

In previous work, *matK* genes have been used to solve the taxonomic relationships of the genus *Elaeagnus* (Cheng et al. 2022). However, a robust phylogeny of this genus is still lacking. Therefore, we constructed a phylogenetic tree based on the chloroplast genome to address this gap. The chloroplast genome assembled here exhibited similarities in size, structure, and gene content to previously reported *Elaeagnus* species, suggesting conservation of the *Elaeagnus* chloroplast genome (Cheng et al. 2020; Lu et al. 2022). The phylogenetic analysis, performed using a maximum-likelihood phylogenetic tree, supported that *E. oldhamii* was clustered to *E. glabra* and *E. macrophylla*. In this study, we unraveled chloroplast genetic makeup of the *Elaeagnus* species, which could be further utilized in population genetic and phylogeographic analysis, thus contributing to the promotion of its species protection. Moreover, the revealed chloroplast genetic makeup could serve as a molecular marker for future studies on hybridization, gene flow, and adaptation in *Elaeagnus* species. These findings offer valuable genetic reservoirs for exploring genetic diversity and further evolutionary relationships within the genus *Elaeagnus*.

## Supplementary Material

Supplemental Material

## Data Availability

The genome sequence data that support the findings of this study are openly available in GenBank of NCBI at (https://www.ncbi.nlm.nih.gov) under the accession no. OQ948125. The associated BioProject, SRA, and Bio-Sample numbers are PRJNA970522, SRR24475791, and SAMN35004913 respectively.
