# Supplementary material for: The complete chloroplast genome and phylogenetic analysis of Elaeagnus oldhamii (Elaeagnaceae) from Fujian, southeastern China
Source: Mitochondrial DNA B Resour. 2024 Jan 18;9(1):109–13. doi: 10.1080/23802359.2024.2305399 (PMC10798290; doi:10.1080/23802359.2024.2305399)
Supplement: Supplemental Material [file TMDN_A_2305399_SM4860.docx]

**Supplemental material**

The online material available for this article contains the following:

**Figure S1.** Overall coverage depth of the complete chloroplast genome of *Elaeagnus oldhamii.*

**Figure S2.** Comparison of the borders of the LSC, SSC, and IR regions of five species of *Elaeagnus*.

**Figure S3.** Visualization of genome alignment of the chloroplast genomes of five *Elaeagnus* species. *E. umbellata* was used as a reference by mVISTA. Gray arrows show the position and direction of each gene. The X-axis represents the midpoint of the window, while the y-axis represents nucleotide diversity (Pi). Colored regions represent exon, intron and intergenic spacer (IGS) sequences.

**Figure S4.** Schematic map of the cis-splicing and trans-splicing genes in the chloroplast genome of *E. oldhamii*.


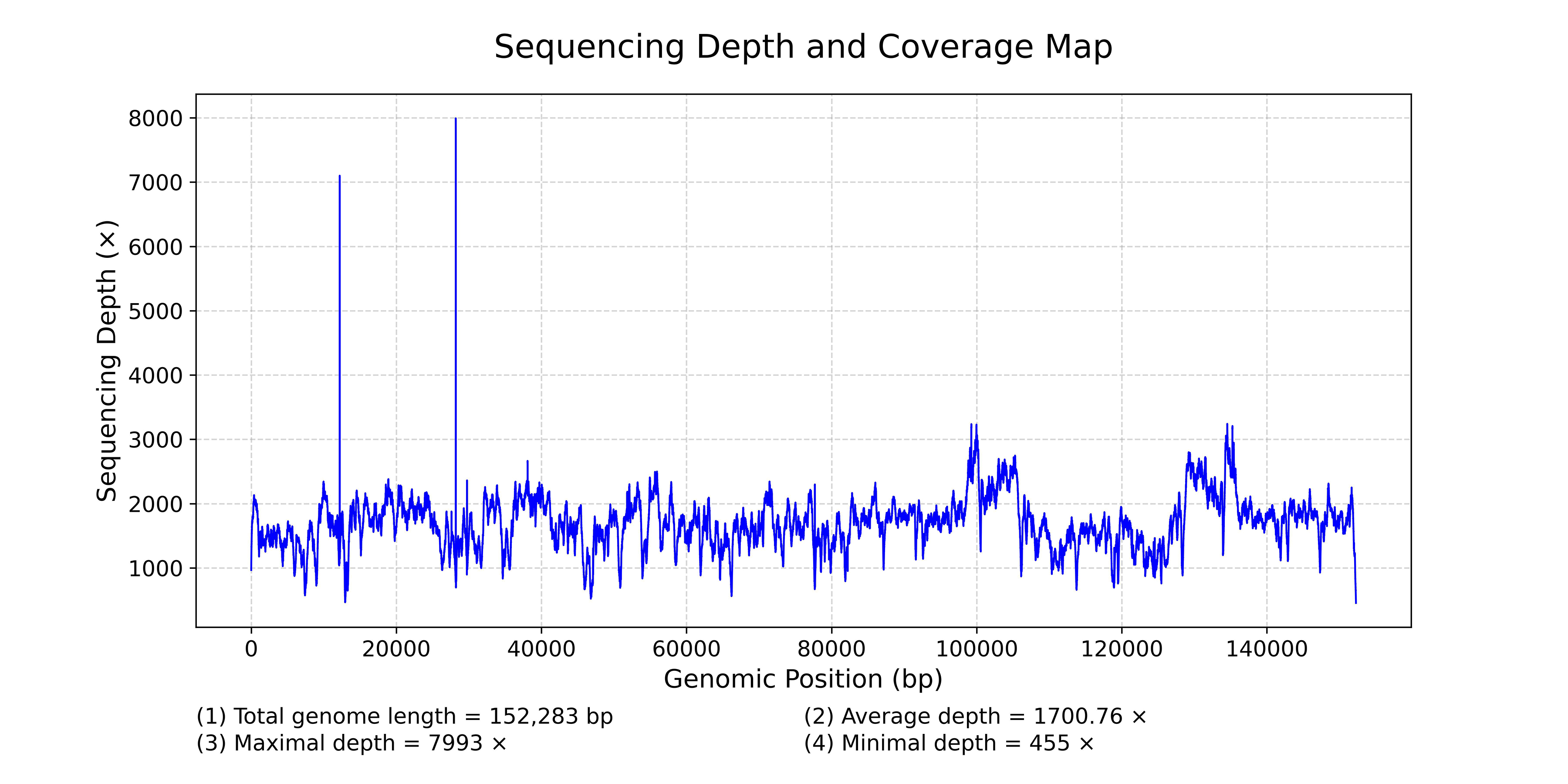


**Figure S1.** Overall coverage depth of the complete chloroplast genome of *Elaeagnus oldhamii*. X-axis and Y-axis respectively represent the base site and mapping depth of the clean data corresponding to the site. The average coverage depth is 1700.76× and the minimal depth is 455×, indicating that the chloroplast genome of *E. oldhamii* was assembled correctly.


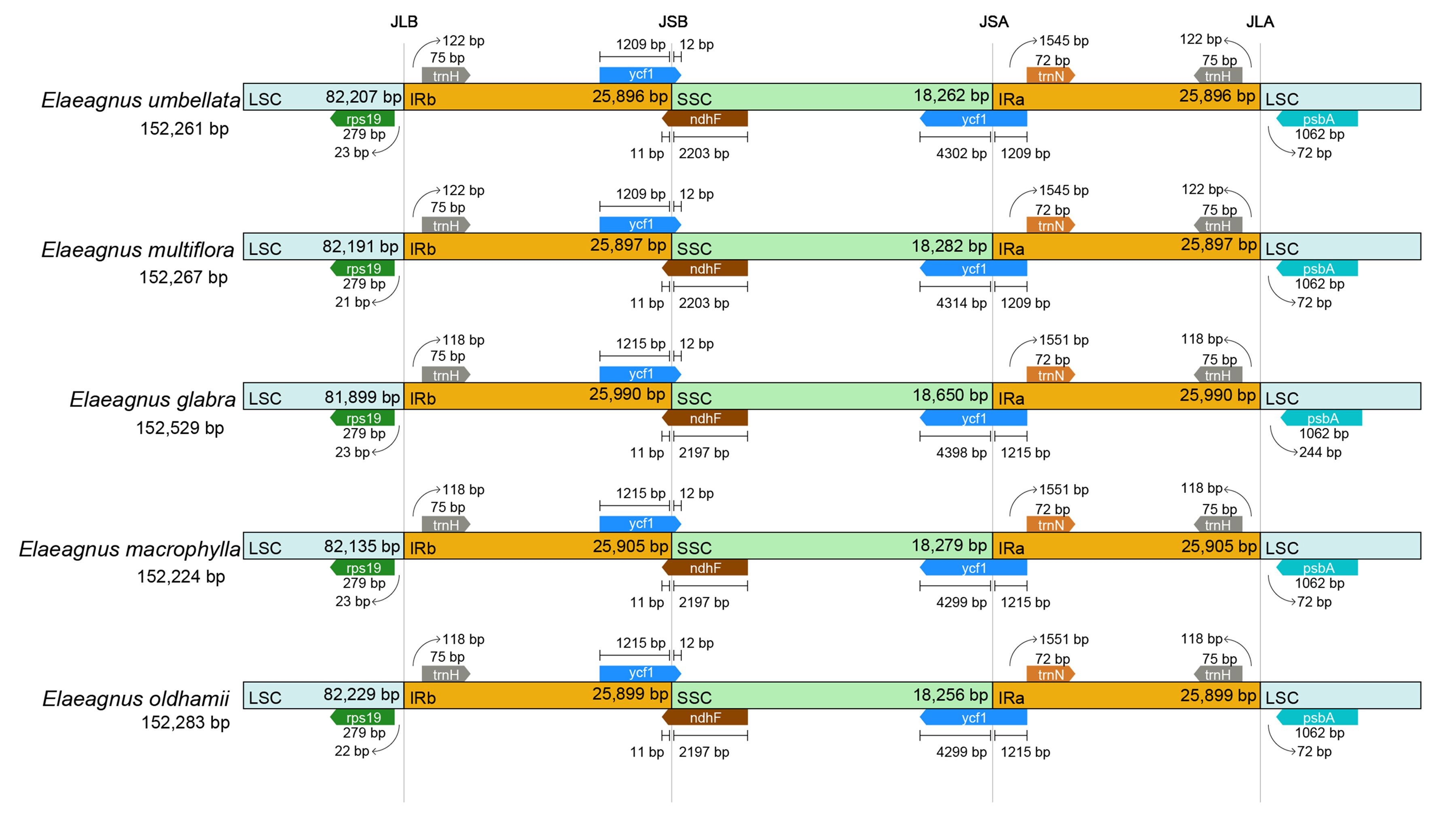


**Figure S2.** Comparison of the borders of the LSC, SSC, and IR regions of five species of *Elaeagnus*. JLA and JLB separate the LSC and IR regions, while JSA and JSB separate the SSC and IR regions. The image shows the length and location of each gene at each boundary.


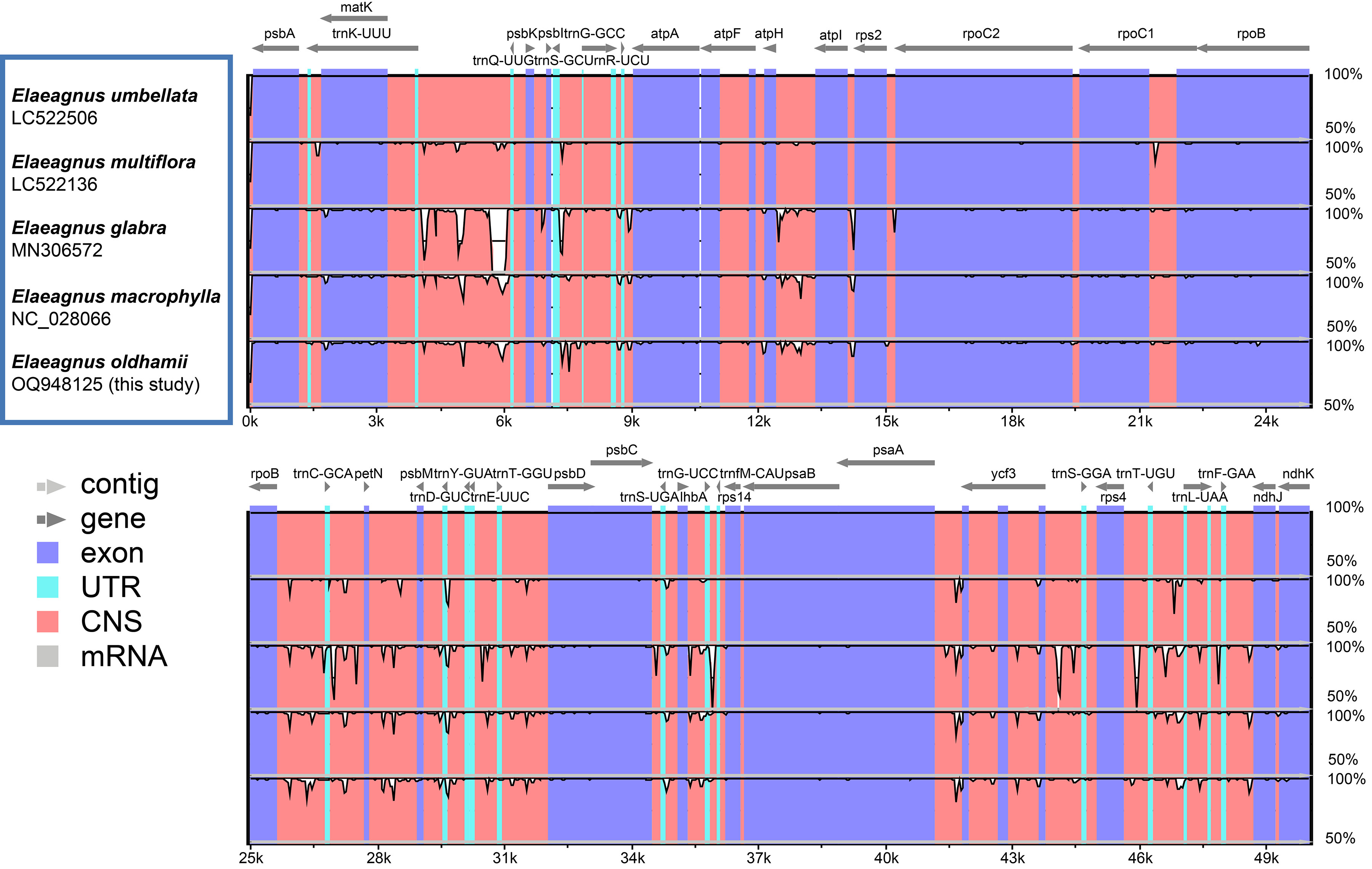


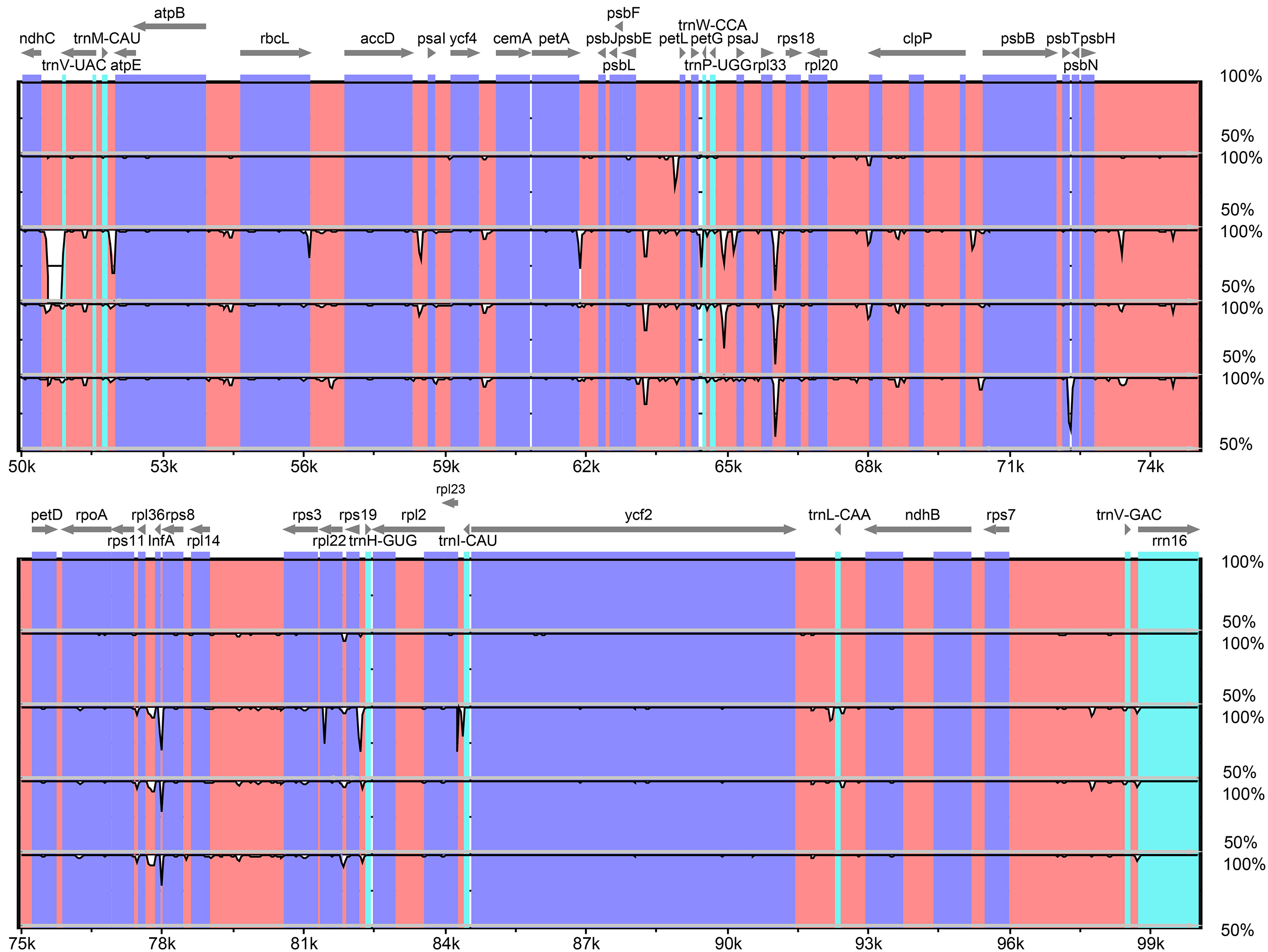


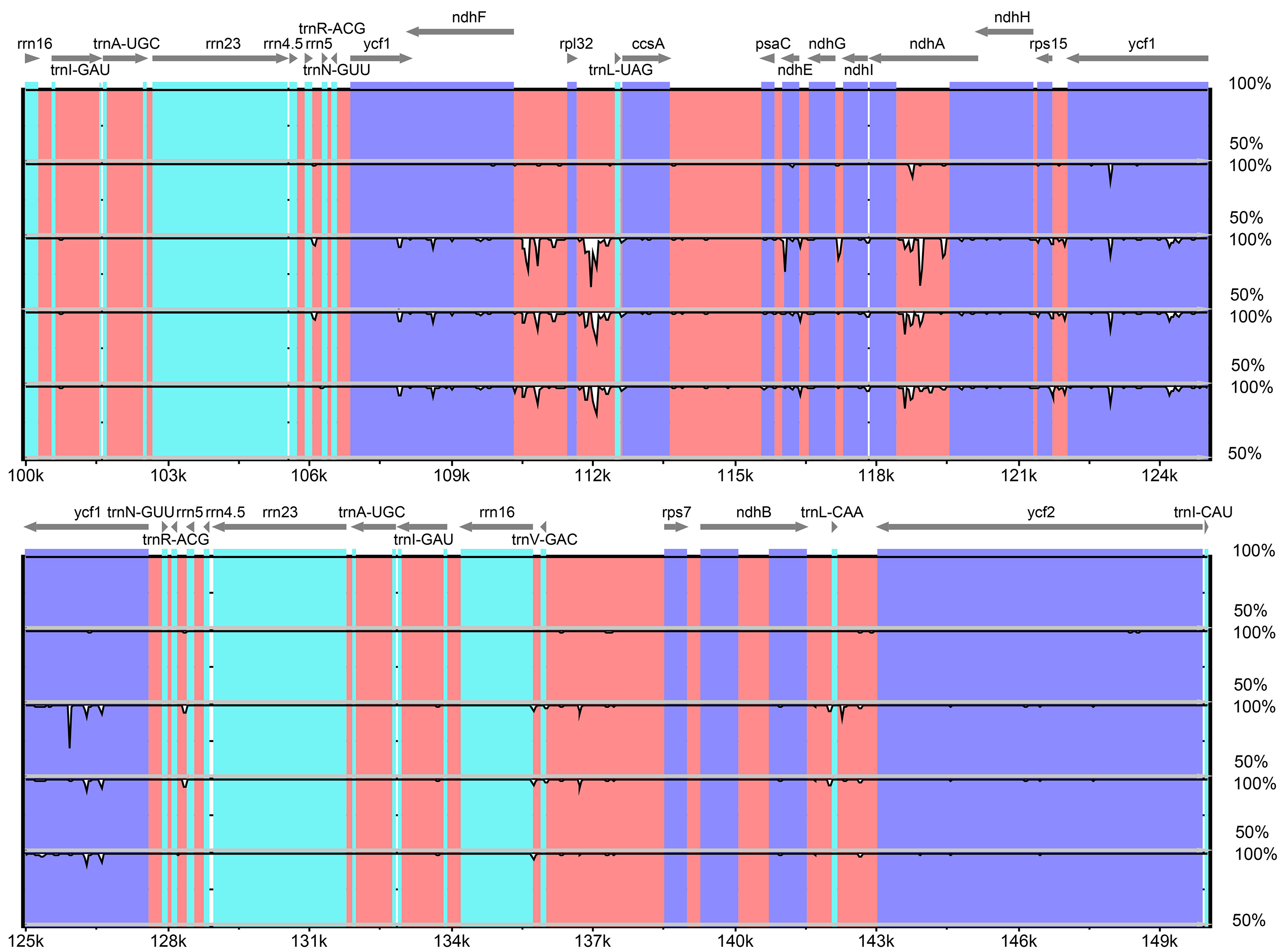


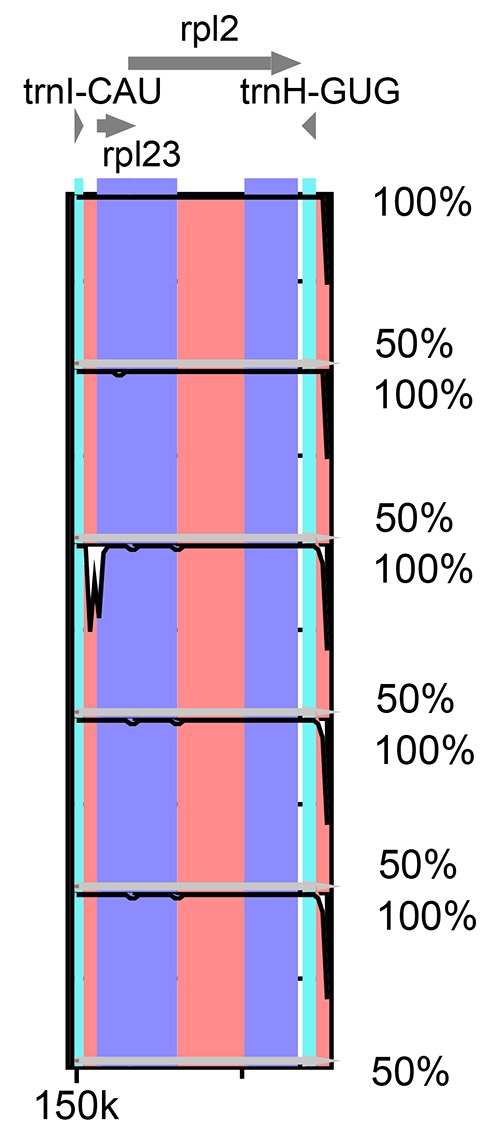


**Figure S3.** Visualization of genome alignment of the chloroplast genomes of five *Elaeagnus* species. *E. umbellata* was used as a reference by mVISTA. Gray arrows show the position and direction of each gene. The X-axis represents the midpoint of the window, while the y-axis represents nucleotide diversity (Pi). Colored regions represent exon, intron and intergenic spacer (IGS) sequences.


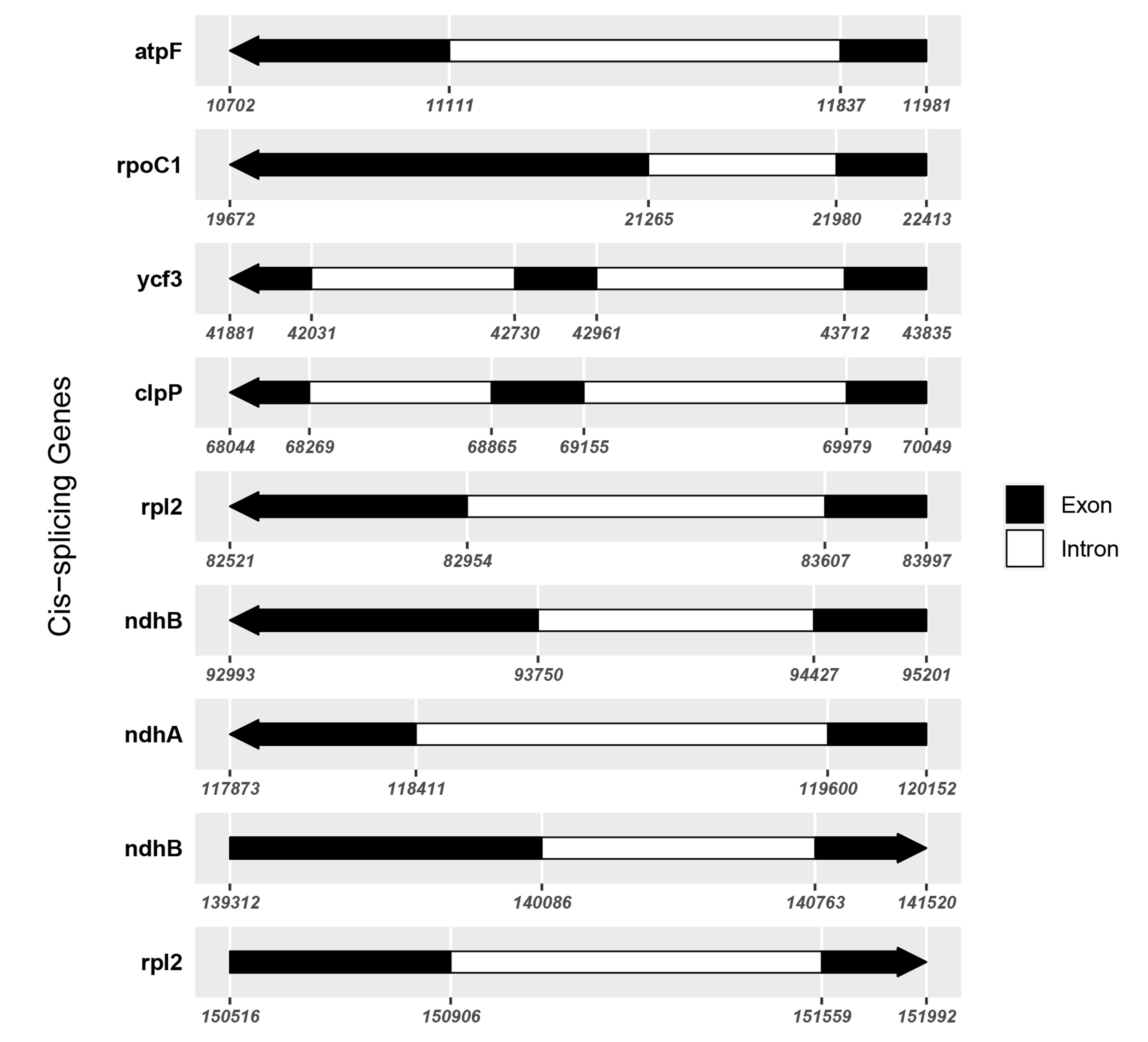

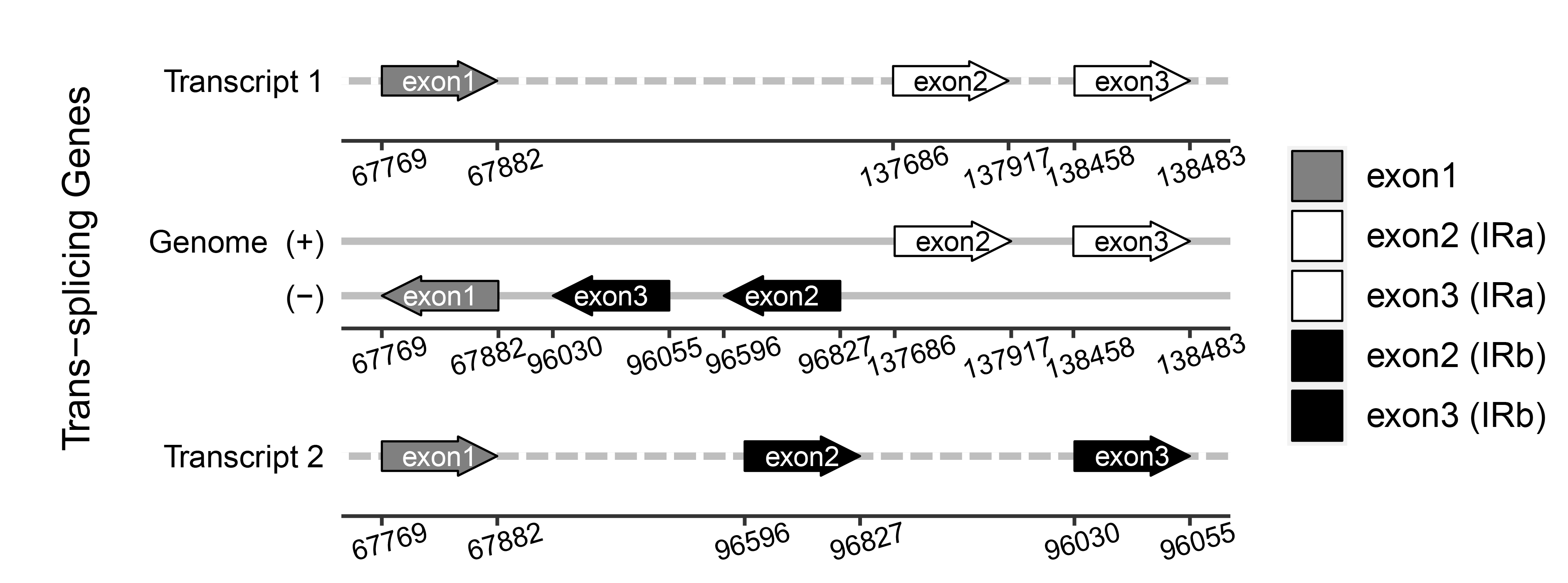


**Figure S4.** Schematic map of the cis-splicing and trans-splicing genes in the chloroplast genome of *E. oldhamii*. Arrows in the figures indicate the direction of genes. The types of nucleotide fragments represented by different colors are shown on the right side of the figures.
